# Ethnobotany in the Cumbres de Monterrey National Park, Nuevo León, México

**DOI:** 10.1186/1746-4269-3-8

**Published:** 2007-01-30

**Authors:** Eduardo Estrada, José A Villarreal, César Cantú, Ismael Cabral, Laura Scott, Carmen Yen

**Affiliations:** 1 Facultad de Ciencias Forestales, Universidad Autónoma de Nuevo León, km 145 Carretera Nacional Linares-Cd. Victoria, A.P. 41, 67700, Linares, N.L., México; 2 Departamento de Botánica, Universidad Autónoma Agraria Antonio Narro, Buenavista, Saltillo, Coahuila 25315, México

## Abstract

An ethnobotanical study in the Cumbres de Monterrey National Park (CMNP), Nuevo Leon, Mexico was conducted. In spite of the large area (1,773.7 km2), heterogeneous physiography, contrasting plant communities and high species diversity of the CMNP, very little was previously known about its useful plants. Based on 95 interviews with inhabitants of the region who were 35 years or older, we recorded ethnobotanical data of 240 species (comprising 170 genera and 69 botanical families), and 146 different uses. Most of the cited uses (98) were found to be medicinal ones.

**Background:**

An ethnobotanical study in the Cumbres de Monterrey National Park (CMNP), Nuevo Leon, Mexico was conducted. In spite of the large area (1,773.7 km^2^), heterogeneous physiography, contrasting plant communities and high species diversity of the CMNP, very little was previously known about its useful plants. Based on 95 interviews with inhabitants of the region who were 35 years old or older, we recorded ethnobotanical data of 240 species (comprising 170 genera and 69 botanical families), and 146 different uses. Most of the cited uses (98) were found to be medicinal ones.

**Methods:**

Ninety five inhabitants 35 years old and oldest were interviewed to know what are the main plant uses in the Cumbres de Monterrey National Park.

**Results and discussion:**

Two hundred and forty species, 170 genera, and 69 families of useful plants and 146 different uses were recorded. We found most of the uses to be medicinal (98), while the rest (48) represent various purposes. Herbaceous plants are the most used, followed by shrubs and trees.

## Background

Northeastern Mexico presents a high diversity of plant associations, including a variety of scrubland types (Tamaulipan, xerophyllous, rosetophyllous and piedmont communities) [1][2][3], chaparral [4], mixed pine-oak forest [3][5], conifer forest [3][6][7][8], halophytic grasslands [9], and alpine meadows [7][10]. Many of these plant communities are currently under threat because of frequent forest fires, deforestation, conversion of natural vegetation into cattle pastures, croplands and fruit orchards, and extensive clearing for human settlements. One of the most significant physiographic units in northeastern Mexico is the Sierra Madre Oriental because of is role in moisture catchment and watershed management, and because it sustains extensive conifer and oak forests, which are the main forest resources of the region. The high plant diversity and scenic beauty of these mountains have merited the establishment of a number of natural protected areas, such as El Cielo Biosphere Reserve, Cumbres de Monterrey National Park, Cerro El Potosí Natural Protected Area and El Tokio Natural Protected Area, among others.

Employment of medicinal or useful plants is a common activity all over Mexico, and, may be closely related to worsening economic conditions [11]. A lack of medical care, unsanitary conditions, malnutrition, and poverty wages [12], together with high cost of medicines had contributed to people's need to use both traditional and modern health care, including medicinal plants. Abundant information about useful plants in Mexico recorded from the mesomaerican tropical south region [13][14][15][16][17][18][19][20] shows the relevance and legacy of the huge knowledge about different plant uses and applications, now widely used in northern areas.

Cumbres de Monterrey is the largest national park in Mexico, covering an area of 177,367 hectares (1,773.7 km^2^). It was established in 1939 by presidential decree to preserve the regional flora and fauna, and today it remains one of the most visited areas in the central part of the State of Nuevo León for picnics, hiking, camping and other recreational activities. The main villages inside the Park are Puerto Genovevo, El Manzano, Ciénega de González, Laguna de Sánchez, El Tejocote, El Hondable, La Camotera, La Trinidad, Potrero Redondo, El Pajonal, El Huajuco, La Huasteca and San Antonio de la Osamenta [21]. The sparse settlements throughout the CMNP reflect contrasting socio-economic conditions of its inhabitants. The ejidos, collectively owned tracts, are made up of low to middle class families who live permanently in the area, while half of the private properties within the Park belong to affluent families who typically do not live there on a permanent basis, but use them only on weekends for leisure. Most of the settlements within the CMNP are located in piedmont scrub and pine-oak forest areas, and only a few of them are present in the driest zone, covered by xerophyllous and rosetophyllous scrub.

The contrasts in topography, climate and soil types within the CMNP determine a heterogeneous vegetation mosaic, each plant community composed at times of hundreds of species. Many of these plants are used by resident people for different purposes: as food, medicine, trade goods, forage, and other uses. Throughout the year, especially on weekends, it is common to see many residents selling their products along the roads leading into the CMNP, especially fresh fruit (peaches, apples, plums, pears, prickly pears, apricots), fruit liquors, candies made out of fruit, breads, medicinal plants, firewood, ornamental plants, etc. Despite its high plant diversity (almost 1,500 plant species and the multiple uses given to the local flora, little information exists regarding human uses of the plants in this area [22][23][24][25]. In more general terms, rather few plant uses have been documented in the State of Nuevo León [26][27][28].

Local residents' knowledge about plant uses in the CMNP has resulted mainly from empirical observation and oral transmission from generation to generation. Much of this traditional knowledge is currently being lost, in large part due to the fact that the younger generations move out of the CMNP to work and study in urban areas, especially in Monterrey. The main objective of the present study is to document the traditional knowledge and uses of plants in this part of northeastern Mexico.

**Figure 1:**
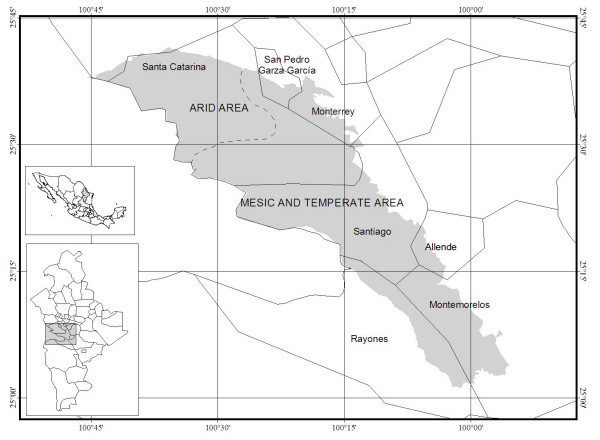


The CMNP is located in the west-central part of the State of Nuevo León, covering parts of seven municipalities: Allende, Montemorelos, Monterrey, Rayones, Santa Catarina, San Pedro Garza García and Santiago (25° 41'-25° 02' N, 100° 45'-99° 11' W) (Fig. 1). Its altitude ranges from 600 to 3,400 m above sea level. The climate is markedly seasonal, with a dry period from November to May, and a wet season from June to October [21][29]. The main climate types present within the Park are semi-warm, temperate-subhumid (mesic and temperate areas, above 750 masl), and arid-warm and very arid-semi-warm (dry areas, below 700 masl), with an annual average temperature of 3° to 18°C in the mesic and temperate areas, and 18° to 22°C in the dry areas, with an annual rainfall between 400 mm in the dry areas and 1000 mm in the mesic and temperate areas [29][30].

## Methods

This study was part of a larger project under the title "Flora of the Cumbres de Monterrey National Park," sponsored by the National Biodiversity Commission (CONABIO, Grant APO-36). All the information regarding plant species, biological forms, habitat, local names and uses was recorded by the authors between April 2004 and April 2006. Ethnobotanical information was obtained through informal interviews conducted during the same period with knowledgeable individuals, ranging in age between 35 and 70 years old. We selected this age group because these generations are most familiar with the different uses of the regional flora. Three quarters of the group were at least 50 years old. All of the people we interviewed belong to the lower income class and do not have permanent jobs, but carry out different kinds of work along the year: cattle ranching, laying bricks, selling plants, etc. All 95 persons interviewed (40 women and 55 men) live permanently in the CMNP; most of them (90) were born in communities within the Park, while the remaining 5 individuals were born in tows nearby (Allende, Montemorelos and Santiago). All the interviews were performed in Spanish, and the informants did not belong to any other linguistic or ethnic group.

We asked our informants which species of plant are used for specific and for general purposes. We took notes during all conversations. No information that was provided to us was left unrecorded, and we have included in this publication all the names and uses of plants that we documented. A voucher specimen of each plant species we collected has been deposited together with all the relevant data in the herbaria CFNL (Linares, N.L.) and ANSM (Saltillo, Coahuila).

Plants collected in the field were brought to the settlements and shown to the people living there to inquire about the common names and uses of each species. In addition, we made frequent field trips with our informants for *in situ *identification of plants they mentioned in the interviews. Given that the CMNP includes two heterogeneous, well differentiated climatic areas, the temperate zone (which is mountainous and covers 74% of the total area), and the arid zone (which occurs in inland basins that cover 26% of the Park), we carried out 65 interviews in the temperate zone and 30 the arid zone. Except for *Cannabis sativa *ssp. *indica *and *Lophophora williamsii *(we took photographic records of both species) which we found preserved in an alcohol solution at doña Anselma's house, voucher specimens of all the species recorded in this study were collected by the authors.

## Results and discussion

We recorded 240 species, 170 genera, and 69 families of plants that grow in the CMNP and have at least one use in the local folk culture. 211 species were recorded in the temperate zone, and 105 in the arid zone, while 74 species grow in both areas. Almost all plants in both climatic zones have only one common name except for two species, *Berberis gracilis *var. *madrensis *(called cuasia and palo amarillo), and *Geranium seemanii *(geranio and alfilerillo). Most of the *Agave, Pinus, Quercus *and *Euphorbia *species are called maguey, pino, encino and golondrina, respectively. Most of the *Mammillarias *are called chilitos, all species of *Chenopodium *and *Teloxys *are known invariably as epazote, and orquídea is applied to all species in the family Orchidaceae. On the other hand, in a few cases the same common name is applied to different genera of the same family, such as poleo (*Agastache *and *Hedeoma*) and yerba de la gallina (*Commelina, Gibasis *and *Tradescantia*), or even to genera in different families, such as siempre viva (*Selaginella*, *Sedum *and *Echeveria*) and salvia (*Salvia *and *Croton*).

A total of 146 different uses were recorded, most of them (98) falling under a broader category of medicinal utility, while the rest (48) refer to various purposes, mainly for human consumption, fodder for domestic animals, firewood, construction materials, live fences, etc. [see Additional file 1]. Most of the information regarding the use of cultivated plants as home remedies was obtained from women, while most of the data on non-medicinal uses of wild plants were provided by older men. In general, most of the women we spoke to stay at home most of time, where the main activities are cooking, taking care of the children, tending gardens of edible and ornamental plants, etc., so they are most familiarized with cultivated plants and their uses, while men carry out field activities such as taking care of livestock, cutting firewood for home use and for sale, sowing plants, collecting fodder for the animals, etc., so they are in closest contact with wild plant and their uses. However, one particular lady known as doña Anselma, one of the most important *matronas *(midwives) and *curanderas *(healers) in the region, has a notable empirical knowledge of the uses of both cultivated and wild plants, and is widely recognized as a reliable healer. Many of the locals prefer to visit her rather than the physicians working at the modern health clinics within the CMNP, and this is partly due to the fact that besides her comprehensive knowledge of plant uses, she has had some academic training as well, having taken a childbirth assistance course.

People in the group from 50 to 75 years of age or older gave us information on 95% of the total plant uses, while individuals 35 to 49 years old provided information on 45% of the plant uses. As a general rule, younger residents in the area know much less about the uses of plants than older people.

The families with the highest number of genera used in the local folk culture are the Asteraceae (14), Cactaceae (12), Fabaceae (11), Rosaceae (8), Lamiaceae (7), Agavaceae (6) and Caesalpiniaceae, Mimosaceae, Solanaceae and Orchidaceae, each of which we found to have five useful genera. On the other hand, the families with the most numerous useful species in the region are the Cactaceae (25), Lamiaceae (16), Asteraceae (15), Agavaceae (12), Fabaceae (11), Rosaceae (10) and Euphorbiaceae and Mimosaceae, with eight useful species each. The families with a higher number of uses are the Asteraceae (41), Lamiaceae (38), Cactaceae (37), Agavaceae (21), Mimosaceae (20), Rosaceae (18), Fabaceae (15), Euphorbiaceae (14), Caesalpiniaceae (9), Crassulaceae (8), Solanaceae (8) and Commelinaceae (6). The species with the most diversified uses are *Aloe vera *(12), *Marrubium vulgare *(8), *Agave bracteosa *and *Zea mays *(7), *Tagetes lucida *and *Allium sativum *(6), and *Acacia farnesiana, Litsea glaucescens, Matricaria recutita, Machaeranthera psamophila, Mirabilis jalapa, Mentha spicata *and *Smilax bona-nox*, *Ageratina *sp.*, Artemisia mexicana, Teloxys ambrosioides, Cupressus arizonica, Ebenopsis ebano, Lantana macropoda, Opuntia *sp., *Prosopis glandulosa *var. *glandulosa, Rosa serrulata, Rosmarinus officinalis, Ruta graveolens *and *Taxus globosa *(5).

### Homegardens

We found 59 species cultivated in local gardens for various purposes, 20 of which grow wild in the area. All the women we interviewed cultivate at least three plant species in their gardens for various uses; the most common species in these gardens are *Mentha spicata, Pelargonium odoratissimum, Ficus carica, Ruta graveolens, Punica granatum, Aloe vera, Foeniculum vulgare, Matricaria recutita, Agave *spp., *Opuntia *spp. and *Hedeoma drumondii*. The exchange of cultivated plants between households, especially those used medicinally or as ornamentals, is a common practice among women living inside the Park.

### Type of use

Out of the 98 medicinal uses of plants we recorded, the most common are the control of colic (21), diabetes (19), stomachaches (9) and headaches (8); in all these cases, plants are boiled and ingested orally. Infusions play a very important role in local folk medicine: they represent the main type of remedy against various kinds of illness in both climatic areas, the temperate and the arid zones. Most infusions are prepared boiling the leaves, flowers and parts of the stems. In most cases, a single plant is used, but in a few instances, two or more species are mixed together to brew a tea. Even though they trust the efficacy of traditional remedies, some people we spoke with, especially those who live near a health clinic or are in close contact with modern medicine, combine empirical and scientific knowledge to treat their maladies (headaches, stomachaches, colic, etc.), and they will drink an herbal infusion along with two aspirins, tylenols, *mejoral *pills or other commercial medicines. They told us, however, that pills can only control the pain, whereas infusions will cure the illness, though they also said that both kinds of remedies are sometimes needed for a sick person to really get well.

Most of the people we interviewed (65) dry and store their medicinal plants to use them later; the dried parts are mostly the leaves, stored in paper bags or hung in bundles from the ceiling. Two common medicinal species, *Lophophora williamsii *and *Cannabis sativa *ssp. *indica*, are usually kept in alcohol (70–90%), since these plants cannot be cultivated, as they are forbidden by law, however, to avoid any type of punish for the government authorities, these plants must be kept in alcohol and also in small quantities. Wild and cultivated edible plants are an important part of the diet of people living in the region. Thirty nine different species are used for this purpose, foremost among them the plants (21 species) that provide fruit, whether it is eaten raw or cooked, belonging to the genera *Echinocereus*, *Cucurbita, Sechium, Persea, Morus, Ficus*, *Punica*, *Crataegus*, *Fragaria*, *Prunus*, *Lycopersicon *and *Physalis*. Fruit plants are followed in importance by species (8) with edible leaves, which belong to the genera *Amaranthus, Rorippa, Tillandsia, Chenopodium, Teloxys, Rumex *and *Portulaca*; plants (8) that bear edible seeds or stems (*Opuntia, Pisum, Vigna, Allium, Ebenopsis *and *Avena*); and species (2) with edible flowers (*Yucca*).

A focus on local use, i.e., the tending of different kinds of plants in a home garden to fulfill the family's needs, is more prevalent in households of the temperate area than in the arid zone. In the former area, some residents harvest much of their own food in their gardens; we recorded 53 domestic units that show different combinations of medicinal, food, beverage, flavoring, firewood and ornamental species. The most common combinations of useful plant were medicinal-food and medicinal-firewood, followed by medicinal-ornamental. In most of the 95 households we visited for our interviews, we recorded several combinations of uses. In most of these gardens, people harvest tomatoes (*Lycopersicon esculentum*), peas (*Pisum sativum*), prickly pears (*Opuntia ficus-indica*) and celery (*Apium graveolens*) for food; chamomile (*Matricaria recutita*), rue (*Ruta graveolens*), mint (*Mentha spicata*) and aloe (*Aloe vera*) for medicine; maguey (*Agave asperrima*) for a beverage and a syrup; fennel (*Foeniculum vulgare*) and basil (*Ocimum basilicum*) as flavoring herbs; pitch pine (*Pinus teocote*) and oaks (*Quercus *spp.) as firewood, and palo casita (*Cornus florida*), chilitos (*Mammillaria *spp.) and siempreviva (*Sedum *spp. and *Echeveria strictiflora*) as ornamental plants.

### Economic botany

Besides these uses, people living in the temperate zone have developed a small industry canning the fruit (conservas) of cultivated species, mainly peaches, apples, pears, apricots and plums. They also produce fruit liqueurs and a beverage obtained from an agave (aguamiel). To make conservas, cultivated fruits are boiled in water and sweetened; after boiling, fruits are immediately packed in glass flasks. The canned fruit keeps well for a long time, and local people sell it throughout the year. Homemade sweet fruit liqueurs (apple, pear and capulín) as well as sotol (a distilled liquor made from *Dasylirion *sp.) have a high demand from Park visitors. Aguamiel is obtained scraping a cavity 15–20 cm in diameter in the center of a mature maguey; once a day, the sap that gathers in the cavity is collected with a cup and refrigerated in plastic containers to be sold. At times, aguamiel is boiled slowly for 3–4 hours to prepare a syrup, a more elaborate product that fetches a higher price. Conservas, fresh cultivated fruits, aguamiel, liqueurs and agave syrup are the main products sold by CMNP residents along the seasons. Except for wild Cactaceae (*Mammillaria *spp., *Echinocereus *spp., *Ariocarpus retusus *and *Astrophytum capricorne*), Crassulaceae (*Echeveria strictiflora*) and Agavaceae (*Agave victoriae-reginae *and *A. bracteosa*) that are offered for sale as ornamentals, people living in the arid zone do not sell any local products as a means of subsistence. However, they do consume various cactus fruits, especially those of *Echinocereus *(pitayas) and *Opuntia *(tunas) species.

Several plants are widely used as animal fodder: residents of the temperate zone who own livestock, mainly bovines, commonly feed them with wild species such as *Opuntia engelmannii *(stems)*, Dalea bicolor *(leaves)*, Eysenhardtia texana *(leaves)*, Quercus *spp. (leaves and acorns)*, Acacia *spp. (leaves), *Leucaena *spp. (leaves) and *Prosopis glandulosa *(leaves and pods). In addition, three cultivated plants are used extensively for this purpose:*Medicago sativa*, *Vicia villosa *and *Opuntia ficus-indica*. *Medicago sativa *is not widely cultivated in the area, but people buy it in the nearest town, Santiago. In recent years, *Vicia villosa *is being cultivated as forage in some of the most productive agricultural areas within the CMNP, such as Laguna de Sánchez. *Plantago major *is a weedy species that grows very commonly on fallowed plots in the highland valleys; it is widely used to feed horses and donkeys, and people harvest it in the fields to take home to feed their animals. Pigs are mainly fed acorns (*Quercus *spp. fruits), which local people consider to be excellent fodder, allowing swine to grow large and healthy, improving the profits when they are sold. The most common prickly pear that is exploited as animal feed is *Opuntia ficus-indica*; since this species lacks prickles, all that people need to do is cut the pads into smaller parts and give them to the cattle. Residents of the dry zone take their livestock (mainly goats and cows) to graze in the mezquite (*Prosopis glandulosa *var. *glandulosa*) bottomlands, where they feed on the pods and leaves, as well as on wild prickly pears (*Opuntia engelmannii*). On cultivated tracts, once corn (*Zea mays*) has been harvested, the dried stems with leaves and tassels are gathered and stored in packs to feed cattle during the winter season. *Echinocactus platyacanthus *(biznaga burra) plays an important role at times of severe drought because it is used as a source of water and food for livestock, mainly goats: residents simply slice up the plants and feed them to the animals.

### Ornamental plants

The ornamental use of wild and cultivated plants is seen more frequently in the temperate area, where the higher diversity and prevalence of appealing herbaceous, shrubby and arboreal life forms have attracted the attention of residents, who have taken many of those species into their gardens as ornamentals for their own enjoyment and also, in few cases, to propagate and sell them in small scale operations. The ornamental plants that are cultivated most commonly in the Park are *Ficus carica, Punica granatum, Acer negundo, Echeveria strictiflora, Begonia gracilis, Mammillaria *spp., *Agave bracteosa, Lonicera pilosa, Sambucus mexicana, Cupressus arizonica, Persea americana, Carya ovata, Rosmarinus officinalis, Litsea glaucescens, Aloe vera, Pteridium aquilinum, Prunus persica, Lantana camara *and *Vitis berlandieri. Echeveria strictiflora *(siempreviva) and *Agave bracteosa *are the wild plants most frequently sold as ornamentals in the CMNP. Some orchids, especially *Dichromanthus cinnabarinus *and *Malaxis macrostchya*, are also used as decorative plants, but are less common. Most of the ornamental species in the arid zone are cultivated, such as *Aloe vera, Bougainvillea glabra, Rosmarinus officinalis, Punica granatum, Ficus carica, Opuntia ficus-indica, Marrubium vulgare *and *Mentha piperita*, but some wild plants are also used for this purpose, such as *Agave bracteosa, Yucca carnerosana *and *Chilopsis linearis*. The epiphyte *Tillandsia usneoides *is collected mainly from *Quercus *spp. trees in the mountains during November and December to be used as a decoration under Christmas trees; it is compressed into packs that weigh 15 to 20 kg to be transported and sold in Monterrey. In spite of the huge quantities available of this species and its easy marketing, few people collect this plant today because of the low price paid for it: 50 centavos (0.5 pesos), approximately equivalent to 5 cents US cy, for 1 kg. Buyers are very picky and will only pay even that low a price for packs where the stems remain green, not if they have turned grey. Currently year, only 5 persons are dedicated to gather it, each one collect approximately 700–1000 kg during the two months of that activity, which represents less than 0.01% of the total resource of this plant, in such a way, its populations have a sustainable management.

Despite its proximity to Monterrey (25 to 50 km away), which is one of the most modern and industrialized cities in Mexico, most of the permanent residents in the ejidos and small villages in the CMNP, who are the poorest inhabitants of the Park, still conserve part of their traditions concerning the uses of wild and cultivated plants. Undoubtedly, a good part of their ethnobotanical knowledge has been lost during the last 100 years. Elderly residents told us that their parents were more knowledgeable about plants since their survival depended on them, and they consider they know only a small fraction of what their parents did. They also commented on how the younger generations are not very interested in the different uses of plants, as they prefer to go to the nearest cities to study or to look for a job. At present, the local folk knowledge of plant uses is vanishing, not only because urbanization and modernity are unavoidably encroaching on the CMNP, but also because young people are migrating away, looking for new life perspectives outside of the Park. For them, knowledge about plants gets reduced to asking their parents for a beautiful plant in the temperate zone to take as a gift for a friend in the city, or where to find *peyote *in the dry zone for an aunt in Monterrey who suffers from arthritis.

Twelve percent of the useful plants reported for the semiarid and temperate areas of Tamaulipas [31] are found in CMNP, especially shrubs and herbaceous species used for medicines, food and fuel. In addition, CMNP has strong affinities in species diversity as well as plant uses with the arid central region of Mexico, where 206 useful native shrub species [32] (in 109 genera and 42 families) have been reported, with more than 220 different uses. Of these, the most diverse families are the Leguminosae (52 species), Fagaceae (*Quercus*, 15) and Asteraceae (12), and the main uses are for medicines, construction, textiles and food. The two areas share 76% of families, 49% of genera, and 44% of species. The genera with similar uses are, for construction: *Dasylirion, Cupressus, Parkinsonia, Acacia, Ebenopsis, Prosopis, Quercus, Pinus *and *Abies*; for medicinal use: *Rhus, Artemisia, Acacia, Tecoma, Arbutus, Salvia, Buddleja, Cowania, Prunus, Salix, Taxodium *and *Larrea*; for food: *Echinocactus, Ebenopsis, Carya, Crataegus, Prunus *and *Celtis*; for fuel: *Cupressus, Parkinsonia, Acacia, Ebenopsis, Mimosa, Prosopis, Quercus, Pinus *and *Taxodium*; for ornamental use: *Bauhinia, Ebenopsis, Pinus, Salix *and *Taxodium*; and for fodder: *Acacia, Leucaena, Prosopis, Eysenhardtia *and *Quercus*. In a similar way, several studies carried out in southern Mexico reveal the nation's rich plant diversity and the extensive knowledge of useful plants. In the State of Hidalgo, 101 species are used for multiple purposes, especially for medicines (47%) and food (22%) [33]. In the Tehuacán-Cuicatlán Valley (Puebla), 49 species of medicinal plant [34] have been recorded in 48 genera and 28 families, most of them wildflowers (48%), 28% in cultivation, and 24% acquired in markets. Almost all of these species (90%) are ingested as an infusion. Most of the cultivated plants used in this area are present and used for similar purposes by inhabitants of the CMNP, mainly those belonging to the genera, *Aloe, Opuntia, Mentha, Ocimum, Prosopis, Psidium, Bouganvillea *and *Ruta*. Along the coastline of Oaxaca, useful plants are used as medicines (80 species) and for construction (19), wood (19) and forage (5), and most commonly belong to the Asteraceae, Leguminosae, Poaceae, Solanaceae, Cucurbitaceae, Rutaceae and Bignoniaceae [35]. In Quintana Roo, 83 useful species have been recorded, most of them arborescent species [36]. The main uses are for medicine, construction, food and fuel, and the main families are the Leguminosae, Rubiaceae, Verbenaceae and Sapotaceae.

As in other regions of Mexico, the main uses of plants in CMNP are for medicines, food, construction and ornaments. People inhabiting the CMNP use herbaceous plants as medicines and food in the same way they do in other regions of Mexico; shrub species are mainly used for fuel, food and ornaments, while arborescent species are used mainly for construction and fuel. Only a few wild arborescent species produce edible fruits, in contrast to the larger number of edible fruits reported for southern Mexico [37].

One of the striking differences between southern Mexico and the CMNP is the number of arborescent species with multiple uses. CMNP has a low diversity and fewer uses of such species than southern Mexico. Arborescent species in CMNP represent only 11% (27 species) of useful plants, while in some areas of southern Mexico, a third or more are arborescent with multiple uses. Arborescent species in CMNP are mainly used for fuel and construction, while 54% of arborescent plants in some areas of Mexico are used for at least 75 different purposes, especially for medicines, construction, food, fencing, fuel, industry, wood, oils, psychotropic substances, dyes and handcrafts [36]. Arborescent legumes play an important role as edible plants, 54% of 86 species recorded in southern Mexico being used for this purpose, especially species of *Acacia, Caesalpinia, Enterolobium, Erythrina, Inga, Leucaena, Pithecellobium *and *Prosopis *[38]. Most of these genera are used as fodder in CMNP. In the La Mancha Natural Reserve, Veracruz, 69% of the arborescent species are useful, mainly being used for construction, fencing, fuel, medicines and ornamental purposes [39].

## Supplementary Material

Additional File 1 Table 1. List of plant species used in the Cumbres de Monterrey National Park, Nuevo León, Mexico. Click here for file
