# Supplementary material for: Ethnobotany in the Cumbres de Monterrey National Park, Nuevo León, México
Source: J Ethnobiol Ethnomed. 2007 Jan 30;3:8. doi: 10.1186/1746-4269-3-8 (PMC1805743; doi:10.1186/1746-4269-3-8)
Supplement: Additional File 1 — Table 1. List of plant species used in the Cumbres de Monterrey National Park, Nuevo León, Mexico. [file 1746-4269-3-8-S1.pdf]

Table 1. List of plant species used in the Cumbres de Monterrey National Park, Nuevo León, México.

Scientific name and voucher specimen number	Common name	Uses	Parts used	Method of use
ACERACEAE				
Acer negundo L. (EE 15998)	Maple	Ornamental<br>Firewood	Whole plant<br>Trunk and branches	Planted in home gardens<br>Made into charcoal
ADIANTACEAE				
Notholaena aschenborniana Kl. (EE 16258)	Helecho	Abortive<br>Treatment to eliminate parasites (worms)	Leaves and roots	Ingestion of infusion
ACANTHACEAE				
Anisacanthus quadrifidus Standl. (EE 16548)	Anisacanto	Ornamental	Whole plant	Planted in home gardens
AGAVACEAE				
Agave americana L. (EE 16203)	Maguey	Beverage	Sap (aguamiel)	Mature plant is tapped for its sap
Agave asperrima Jacobi (EE 12750)	Maguey	Beverage<br><br>Alcoholic beverage (pulque)<br>Syrup<br>Food (quiote)	Sap (aguamiel)<br><br>Sap<br>Sap<br>Inflorescence	Mature plant is tapped for its sap<br>Sap is fermented<br>Sap is boiled until thick<br>Baked in a pit oven
Agave bracteosa S. Watson ex Engelm. (EE 12749)	Amole de Castilla	Treatment of internal traumatism/ renal problems,<br>and to detoxify the body<br>Treatment of diabetes<br>To restore receding hair<br>Beverage<br>Fermented beverages (pulque and mezcal)<br>Syrup<br>Food (quiote)	Roots<br><br>Roots<br>Roots<br>Sap (agua miel)<br>Sap<br>Sap<br>Inflorescence	Rubbed on the body /infusion<br><br>Ingestion of infusion<br>Rubbed on the hair<br>Plant is tapped for its sap<br>Sap is fermented<br>Sap is boiled for several hours<br>Baked in a pit oven, sweetened with sugar
Agave lophantha Schiede	Maguey	Fiber	Leaves	Fiber is scraped from the

(EE 16220)				leaves
Agave scabra Ortega (EE 16183)	Maguey	Fodder	Leaves, roots	Fed raw to animals
Agave victoriae-reginae T. Moore (EE 12748)	Maguey	Ornamental	Whole plant	Planted in home gardens
Manfreda maculosa (Hook.) Rose (EE 16130)	Iris	Ornamental	Whole plant	Planted in home gardens
Yucca carnerosana (Trel.) McKelvey (EE 19639)	Palma pita	Food (chochas)<br><br>Live fences	Flowers (removing the gynoecium and androecium)<br>Whole plant	Cooked with garlic and salt<br><br>Planted in a row
Yucca treculeana Carrière (EE 16233)	Yuca	Food (chochas)	Flowers (removing the gynoecium and androecium)	Cooked with garlic and salt
AMARANTHACEAE				
Alternanthera caracasana Kunth. (EE 16559)	Tianguis	Remedy for fever, lowers high temperature	Leaves	Ingestion of infusion with *Borago officinalis*
Amaranthus viridis L. (EE 16200)	Quelite	Food	Tender leaves and stems	Eaten raw or cooked
ANACARDIACEAE				
Rhus virens Lindh ex A. Gray (EE 16543)	Lantrisco	Treatment of diabetes	Bark	Ingestion of infusion
APIACEAE				
Apium graveloens L. (EE 16074)	Apio	Flavoring herb and salad vegetable	Leaves and stems	Mixed with other vegetables
Foeniculum vulgare Mill. (EE 15993)	Hinojo	Remedy for colic<br>Flavoring herb	Leaves<br>Leaves	Ingestion of infusion<br>Macerated
APOCYNACEAE				
Vinca major L. (EE 15977)	Teresita	Ornamental	Whole plant	Planted in home gardens
ASTERACEAE				
Achillea millefolium L. (EE 16155)	Real de oro	Remedy for colic	Stems and leaves	Ingestion of infusion
Ageratina wrightii (A. Gray) R. M. King & H. Rob. (EE 16021)	Ámbula	Remedy for stomachaches and colic<br><br>Treatment of "fright" (a folk illness)	Leaves<br><br>Leaves, stems and flowers	Eaten raw or cooked, or taken<br>as an infusion<br>The whole body is "cleansed" by gently brushing it with the plant on

		To increase the appetite of children	Leaves and stems	several successive days Administered raw or in an infusion
Artemisia ludoviciana Nutt. (EE 19011)	Estafiate	Treatment of diabetes, indigestion, stomachaches and diarrhea	Aerial parts	Ingestion of infusion
Bidens odorata Cav. (EE 16058)	Altamisa	Treatment of menstrual hemorrhages<br><br>Abortive	Leaves and flowers<br><br>Leaves and flowers	Ingestion of infusion with *Ruta graveolens*<br>Ingestion of infusion with *Ruta graveolens*
Chrysactinia mexicana A. Gray., (EE 16099)	Yerba de San Nicolás	Remedy for infertility<br><br><br>Control of urinary incontinence	Leaves and stems<br><br><br>Leaves and stems	Ingestion of infusion with *Vitis berlandieri*, taken orally and as a nocturnal shower<br>Ingestion of infusion
Chrysactinia truncata S. Watson (EE 16478)	Yerba de San Nicolás	Remedy for infertility	Leaves and stems	Ingestion of infusion with *Vitis berlandieri*
Chrysanthemum parthenium (L.) Pers. (EE 16064)	Altamisa	Treatment of urinary problems, headaches and mouth ulcers	Leaves and inflorescence	Ingestion of infusion
Ageratina viburnoides (DC.) R.M. King & H. Rob. (EE 19348)	Yerba del burro	Treatment of hemorrhages	Leaves	Ingestion of infusion with leaves of *Tagetes lucida*
Flourensia cernua DC. (EE 16678)	Hojasé	Control of parasites	Leaves	Ingestion of infusion
Gnaphalium semiamplexicaule DC. (EE 16191)	Gordolobo	Cough remedy<br>To alleviate cough with phlegm<br><br>Remedy for colic<br>Treatment of bronchitis	Leaves, stems and flowers<br>Leaves and stems<br><br>Leaves and stems<br>Leaves	Ingestion of infusion<br>Ingestion of infusion with bee honey<br>Ingestion of infusion<br>Ingestion of infusion with a piece of ocote (*Pinus teocote)*
Gymnosperma glutinosum Less. (EE 11283)	Tata Lencho	Treatment of fractures and broken bones	Leaves	Poultice with *Rosmarinus officinalis* and the root of *Wilcoxia poselgeri*
Machaeranthera scabrella (Greene) Shinners (JV 1252)	Árnica	Remedy for fatigue	Flowers	The body is massaged with the flowers mixed with the pulp of *Lophophora*

		To treat insect bites and to wash wounds<br>Treatment of stomach ulcers of diabetic patients<br>Stomach infections<br>Muscle pain<br>Skin infections and warts	Flowers<br>Leaves and flowers<br>Leaves<br>Leaves<br>Leaves and flowers	*williamsii* and one half of a *Persea americana* seed, fermented for 9 days in 99% alcohol<br>Infusion with *Equisetum laevigatum* is applied on the skin<br>Ingestion of infusion<br>Ingestion of infusion<br>Infusion applied with a cotton wad<br>Infusion applied with a cotton wad
Matricaria recutita L. (EE 16065)	Manzanilla	Treatment of eye infections of infants<br>Colic in infants<br>Chills and catarrh<br>Red eyes<br>To alleviate and eliminate hematomas	Stem, leaves and flowers<br>Leaves<br>Leaves, stems and flowers<br>Leaves, stems and flowers<br>Leaves, stems and flowers	Infusion applied with a wad of cotton<br>Infusion administered as nasal drops<br>Infusion applied to the eyes with a cotton wad<br>Infusion applied with a cotton wad<br>Infusion applied to the wound with a cotton wad
Tagetes lucida Cav. (EE 16098)	Yerbanís	Hot beverage<br>Treatment of scorpion stings<br>Remedy for insomnia<br>Congestion<br>Intestinal cramps<br>Antihelmintic	Aerial parts<br>Leaves, stems and flowers<br>Leaves<br>Leaves and flowers<br>Leaves, stems and flowers<br>Leaves	Ingestion of infusion<br>Ingestion of infusion with *Allium sativum*<br>Ingestion of infusion with *Citrus aurantium* (naranja) peel<br>Ingestion of infusion<br>Ingestion of infusion<br>Ingestion of infusion
Taraxacum officinale (L.) Weber (EE 15953)	Diente de león	Treatment of pancreatitis	Roots	Ground and boiled, ingested as an infusion
BASELLACEAE				
Anredera versicaria (Lam.)	Sacasil	Treatment of fractures	Roots	Poultice

C.F. Gaertn. (EE 16158)		To clean bruises<br>To alleviate and eliminate hematomas	Roots<br>Sap of the roots	Boiled with *Aloe vera*<br>Applied on the hematoma
BEGONIACEAE				
Begonia uniflora S. Watson (JV 8683)	Begonia	Ornamental<br>Vomitive	Whole plant<br>Roots	Planted in home gardens<br>Ingestion of infusion
BERBERIDACEAE				
Berberis gracilis Hartw. ex Benth. (EE 16322)	Palo amarillo, cuasia	Treatment of renal problems<br>Fly repellent	Bark<br>Bark, leaves and stems	Ingestion of infusion<br>Boiled; infusion is sprayed
BETULACEAE				
Carpinus caroliniana Walter (EE 16460)	Olmo	Ornamental	Whole plant	Planted in home gardens
BIGNONIACEAE				
Chilopsis linearis Sweet (EE 16151)	Mimbre	Cough remedy<br>Heart ailments	Flowers<br>Flowers	Ingestion of infusion<br>Ingestion of infusion
Tecoma stans (L.) Kunth (EE 16123)	Tronadora	Treatment of diabetes	Leaves, stems and flowers	Ingestion of infusion
BORAGINACEAE				
Borago officinalis L. (EE 19081)	Borraja	Febrifuge	Leaves	Ingestion of infusion
BRASSICACEAE				
Lepidium virginicum L. (EE 16430)	Lentejuela	Treatment of amoebic dysentery	Whole plant	Ingestion of infusion in milk
Rorippa nasturtium-aquaticum (L).Hayek (EE 16539)	Berro	Food	Leaves	Eaten raw in salads and cooked with eggs
BROMELIACEAE				
Tillandsia recurvata L. (EE 19316)	Lirio	Food	Tender stems	Eaten raw
Tillandsia usneoides L., (EE 19335)	Paixtle	Remedy for hemorrhoids<br>Decoration	Whole plant<br>Whole plant	Ingestion of infusion<br>Spread below the Christmas tree
BUDDLEJACEAE				
Buddleja cordata Kunth (EE 16754)	Tepozán	Remedy for headaches<br>Body aches	Leaves<br>Leaves, stems and flowers	Ingestion of infusion<br>Ingestion of infusion
CACTACEAE				

Ariocarpus retusus Scheidw. (MGB 233)	Chaute	Treatment of body aches and rheumatism	Whole plant	Eaten or ingested as an infusion
Astrophytum capricorne Britton & Rose (MGB 256)	Anzuelo	Ornamental	Whole plant	Planted in home gardens
Cylindropuntia leptocaulis (DC.) F.M. Knuth (MGB 341)	Tasajillo	Treatment of diabetes	Parenchyma	Blended into a beverage with orange juice
Echinocactus horizonthalonius Lemaire, (MGB 411)	Manca caballo	Ornamental	Whole plant	Planted in home gardens
Echinocactus platyacanthus Link & Otto, (MGB 178)	Biznaga burra	Candy<br><br><br>Fodder and drink for domesticated animals	Pulp of the stem<br><br><br>Pulp	Cut in pieces, allowed to dry for 2 days and cooked with sugar<br>Cut in slices and fed raw to animals
Echinocereus dasyacanthus Engelm. (MGB 294)	Pitaya	Ornamental	Entire plant	Planted in home gardens
Echinocereus knippelianus Liebm. (EE 13245)	Peyote	Treatment of bruises and body aches	Parenchyma	Rubbed on afflicted parts
Echinocereus pectinatus Engelm. (MGB 265)	Pitaya	Food	Fruit	Eaten raw
Echinocereus reichenbachii (Terscheck) Britton & Rose ssp. *armatus* (Poselg.) N.P. Taylor (EE 19628)	Pitaya	Food	Fruit	Eaten raw
Echinocereus stramineus Engelm. ex Ruempl. (EE 19632)	Pitaya	Food	Fruit	Eaten raw
Escobaria tuberculosa (Engelm.) Britton & Rose (MGB 373)	Chilitos	Ornamental	Whole plant	Planted in home gardens
Ferocactus hamatacanthus (Muhlenpf.) Britton & Rose (EE 19629)	Anzuelo	Ornamental<br>Food	Entire plant<br>Parenchyma	Planted in home gardens<br>Cooked and sweetened
Ferocactus pilosus (Galeotti ex Salm-Dyck) Werderm.	Biznaga roja	Ornamental<br>Food	Entire plant<br>Fruit	Planted in home gardens<br>Eaten raw

(EE 19505)				
Lophophora williamsii (Lem. ex Salm-Dyck) J.M. Coult. (recorded preserved in alcohol)	Peyote	Remedy for fatigue<br>Treatment of concussions<br>Rheumatism	Stems<br>Stems seeped in alcohol<br>Stems seeped in alcohol	Eaten raw<br>Rubbed on the muscles<br>Rubbed on the afflicted area
Mammillaria chionocephala J.A. Purpus (EE 19625)	Chilitos	Ornamental	Entire plant	Planted in home gardens
Mammillaria heyderi Muehlenpf. ssp. *hemisphaerica* (Engelm.) D.R. Hunt (MGB 286)	Chilitos	Ornamental	Entire plant	Planted in home gardens
Mammillaria heyderi Muehlen. ssp. *heyderi* (MGB 311)	Chilitos	Ornamental	Entire plant	Planted in home gardens
Mammillaria pottsii Scheer ex Salm-Dyck (MGB 378)	Chilitos	Ornamental	Entire plant	Planted in home gardens
Mammillaria prolifera ssp. *texana* (Engelm.) Borg (EE 19627)	Chilitos	Ornamental	Entire plant	Planted in home gardens
Opuntia engelmannii Salm-Dyck (EE 19618)	Nopal	Treatment of diabetes<br><br><br>Fodder	Stems<br><br><br>Stems	Blended as a beverage with orange and lime juice, after prickles are singed off<br>Fed raw to animals
Opuntia ficus-indica (L.) Mill. (EE 19633)	Nopal de Castilla	Glucose control<br><br>Treatment of diabetes<br><br>Fodder<br><br>Food<br>Food (tunas)	Parenchyma of the stems<br><br>Roots<br><br>Stems<br><br>Stems<br>Fruit	Blended as a beverage with orange and lime juice<br>Blended with the roots of *Ricinus communis* and ingested<br>Fed raw to animals after prickles are singed off<br>Cooked<br>Boiled with sugar or eaten raw
Opuntia imbricata (Haw.) DC. Knuth. (EE 19619)	Coyonoxtle	Food	Fruit	Eaten raw (sour prickly pear)

Opuntia phaeacantha Engelm. (EE 19639)	Nopal	Fodder	Stems	Fed raw to animals after prickles are singed off
Opuntia stenopetala Engelm. (EE 19617)	Nopal	Glucose control<br><br>Treatment of diabetes<br><br>Fodder<br><br><br>Food<br>Food (tunas)<br>Canned food	Stems<br><br>Roots<br><br>Roots and stems<br><br><br>Stems<br>Fresh fruit<br>Fruit	Blended as a beverage with orange and lime juice<br>Blended with the roots of *Ricinus communis* and ingested<br>Stems and roots are fed raw to animals after prickles are singed off<br>Cooked<br>Eaten raw<br>Boiled with sugar
Sclerocactus scheeri (Salm-Diyck) N.P. Taylor (EE 16523)	Biznaga	Ornamental	Entire plant	Planted in home gardens
Selenicereus spinulosus) Britton & Rose (EE 16522)		Food<br>Ornamental	Fruit<br>Entire plant	Eaten raw<br>Planted in home gardens
CAESALPINIACEAE				
Bauhinia macranthera Benth. ex Hemsl. (EE 13319)	Pata de vaca	Ornamental	Whole plant	Planted in home gardens
Caesalpinia mexicana A. Gray (EE 13316)	Yerba del potro	Ornamental<br>Useful wood	Whole plant<br>Stems and branches	Planted in home gardens<br>Handicrafts, hand tools
Cercis canadensis L. var. *mexicana* (Rose) M. Hopk. (EE 19301)	Duraznillo	Ornamental	Whole plant	Planted in home gardens
Parkinsonia aculeata L. (EE 16569)	Retama	Construction material<br>Ornamental<br>Fodder	Stems and branches<br>Whole plant<br>Leaves	To build fences<br>Planted in home gardens<br>Fed raw to animals
Pomaria canescens (Fisher) B.B.Simpson (EE 18476)	Chancaca	To alleviate the pain of hemorrhoids<br>Diabetes	Leaves<br>Leaves	Ingestion of infusion<br>Ingestion of infusion
Pomaria melanosticta S. Schauer (EE 19302)	Chancaca	Treatment of hemorrhoids	Leaves	Ingestion of infusion
CANNABACEAE				
Cannabis sativa ssp. *indica*	Marihuana	Treatment of glaucoma	Leaves	Infusion used as eye drops

(Lam) E. Small & A. Cronquist (recorded preserved in alcohol)		Remedy for fatigue<br><br><br><br><br>Arthritis	Leaves (with flowers of *Machaeranthera scabrella* and leaves of *Bidens odorata*, immersed in 99% alcohol, fermented for 9 days)<br>Leaves and flowers	Rubbed on the body<br><br><br><br><br>Massaged on afflicted parts
CAPRIFOLIACEAE				
Lonicera pilosa Willd. ex Kunth (EE 16344)	Madreselva	Ornamental	Whole plant	Planted in home gardens
Sambucus mexicana DC. (EE 17243)	Sauco	Treatment after childbirth<br>Ornamental	Branches<br>Whole plant	Vaginal washings<br>Planted in home gardens
CHENOPODIACEAE				
Chenopodium berlandieri Moq. (EE 18935)	Epazote	Treatment of blood infections<br><br><br>Remedy for asthma and constipation<br>Intestinal parasites	Leaves (with *Artemisia mexicana* and *Lippia graveolens*)<br>Leaves<br>Leaves (with leaves of *Mentha spicata* or milk)	Ingestion of infusion<br><br><br>Nasal drops<br>Ingestion of infusion
Chenopodium incisum Poir. (EE 16554)	Epazote	Treatment of blood infections<br><br><br>Remedy for asthma and constipation	Leaves (with *Artemisia mexicana* and *Lippia graveolens*)<br>Leaves	Ingestion of infusion<br><br><br>Nasal drops
Chenopodium murale L. (EE 16286)	Epazote de olor	Treatment of blood infections<br><br><br>Remedy for asthma and constipation<br>Flavoring herb	Leaves (with *Artemisia mexicana* and *Lippia graveolens*)<br>Leaves<br>Leaves (cooked with *Phaseolus vulgaris*)	Ingestion of infusion<br><br><br>Nasal drops<br>Salads, soups
Teloxys ambrosioides (L.) W.A. Weber (EE 16072).	Epazote	Treatment of blood infections<br><br><br>Remedy for asthma and constipation<br>Antihelmintic<br><br>Flavoring herb	Leaves (with *Artemisia mexicana* and *Lippia graveolens*)<br>Leaves<br>Leaves (with leaves of *Mentha spicata* or milk)<br>Leaves	Ingestion of infusion<br><br><br>Nasal drops<br>Ingestion of infusion<br><br>Cooked with beans

COMMELINACEAE				
Commelina dianthifolia Delile (EE 14839)	Yerba de la gallina	To alleviate pain after childbirth	Whole plant	Ingestión of infusion
Commelina erecta L. (EE 16243)	Yerba de la gallina	To stop bleeding<br>Ornamental	Leaves<br>Whole plant	Ingestion of infusion<br>Planted in home gardens
Gibasis pellucida (M. Martens. & Galeotti.) D.R. Hunt (EE 16296)	Yerba de la gallina	Ornamental	Whole plant	Planted in home gardens
Tradescantia brevifolia (Torr.) Rose (EE 13336)	Verbena, yerba de la gallina	Febrifuge	Leaves, stems and flowers	Ingestion of infusión
Tradescantia pringlei S. Watson (EE 16781)	Yerba de la gallina	Febrifuge	Leaves, stems and flowers	Ingestión of infusión
CONVOLVULACEAE				
Ipomoea pubescens Lam. (EE 14840)	Correhuela	Ornamental	Whole plant	Planted in home gardens
CORNACEAE				
Cornus florida L. (EE 15403)	Palo casita	Black dye	Bark	Boiled in water
CRASSULACEAE				
Echeveria strictiflora A. Gray,(EE 13358)	Siempre viva	Ornamental	Whole plant	Planted in home gardens
Kalanchoe daigremontiana Hamet & Perrier (EE 16704)	Kalanchoe	Anti-inflammatory	Leaves	Poultice
Sedum greggii Hemzl .(EE 16344)	Siempre viva	Ornamental	Whole plant	Planted in home gardens
Sedum madrense S. Watson (EE 16722)	Siempre viva	Treatment of fungal infections of the mouth<br>To remove debris from the eyes	Leaves<br>Leaves	Pulp is rubbed on afflicted area<br>Viscous juice squeezed out of the leaves is used to wash the cornea (debris adheres to the liquid and is drained out of the eye)
Sedum moranense Kunth (EE 12587)	Siempre viva	Ornamental	Whole plant	Planted in home gardens
Sedum palmeri S. Watson	Siempre viva	Ornamental	Whole plant	Planted in home gardens

(EE 13299)				
Sedum papillicaulum G.L. Nesom (EE 16519)	Siempre viva	Ornamental	Whole plant	Planted in home gardens
Sedum praealtum A. DC. (EE 16027)	Siempre viva	Ornamental	Whole plant	Planted in home gardens
CUCURBITACEAE				
Cucurbita ficifolia C.D. Bouché (EE 16396)	Chilacayote	Sweets	Fruit	Cut in pieces and seeped with water and lime (calcium oxide) for 12 hours, then rinsed clean and boiled in water with 2-3 kg of sugar and apple (*Malus domestica*) twigs
Cucurbita foetidissima Kunth (EE 16398)	Calabacilla loca	Shampoo<br>Food	Fruit pulp<br>Flowers	Hair wash<br>Cooked with cheese
Cucurbita sp. (EE 16943)	Calabacilla	Detergent	Fruit	Boiled and used as soap
Sechium edule (Jacq.) Sw. (EE 18967)	Chayote	Food	Fruit	Eaten cooked, in salads or soup
CUPRESSACEAE				
Cupressus arizonica Greene (EE 11809)	Cedro	Remedy for stomachaches<br><br>To control flatulence<br>Firewood<br>Construction material	Bark and leaves (with *Bidens odorata*)<br>Bark and leaves<br>Trunk, branches<br>Trunk, branches	Ingestion of infusion<br><br>Ingestion of infusion<br>Burnt in kitchen fires<br>Fences; beams and rafters for house roofs
Juniperus deppeana Steud. (EE 11282)	Táscate	Firewood<br>Construction material	Trunk and branches<br>Trunk and branches	Burnt in ovens<br>Fences, beams and rafters for house roofs
Juniperus flaccida Schltdl. (EE 12741)	Táscate	Firewood<br>Construction material	Trunk and branches<br>Trunk and branches	Burnt in ovens<br>Fences, beams and rafters for house roofs
EBENACEAE				
Diospyros texana Scheele (EE 16252)	Chapote	Treatment of diabetes	Leaves	Ingestion of infusion
EQUISETACEAE				
Equisetum laevigatum A.	Cola de caballo	Remedy for renal ailments	Stems	Ingestion of infusion

Br. (EE 16079)		Prostate problems	Stems (with *Zea mays* flowers) styles, called "corn hairs," and the calyx of *Physalys staphelioides*)	Ingestion of infusion
ERICACEAE				
Arbutus xalapensis Kunth. (EE 14791)	Madroño	Mouth wash	Fruit	Chewed raw
EUPHORBIACEAE				
Acalypha lindhemeri Mull. Arg. (EE 16080)	Yerba del cáncer	To wash wounds<br>To alleviate the pain due to traumatism	Leaves<br>Stems and leaves	Infusion applied to wound<br>Ingestion of infusion
Acalypha monostachya Cav. (EE 15966)	Yerba del cáncer	To wash wounds<br>To alleviate the pain due to traumatism	Leaves<br>Stems and leaves	Infusion applied to wound<br>Ingestion of infusion
Croton torreyanus Mull. Arg. (EE 16553)	Salvia	Treatment of anemia	Leaves (with *Salvia regla* or *Salvia greggii*)	Ingestion of infusion
Euphorbia astyla Engelm. ex Boiss. (EE 16750)	Golondrina	Remedy for hemorrhoids	Leaves	Ingestion of infusion
Euphorbia dentata Michx. (EE 16564)	Golondrina	Dandruff	Leaves and stems	Hair washed with a decoction two or three times a week
Euphorbia fendleri(Torr. & A. Gray (EE 16678)	Golondrina	Hemorrhoids	Leaves	Ingestion of infusion
Euphorbia graminea Jacq. (JV 8588)	Golondrina	Hemorrhoids	Leaves	Ingestion of infusion
Euphorbia hyssopifolia L. (EE 16693)	Yerba de la golondrina	Hemorrhoids<br>Treatment of acne	Leaves<br>Stems and leaves	Ingestion of infusion<br>Face washed with an infusion twice a week
Tragia ramosa Torr. (EE 18921)	Ortiguilla	Remedy for the flu and constipation<br>Headaches	Stems<br>Leaves	Ingestion of infusion<br>A raw leaf is placed on the skin on each side of the head
FABACEAE				
Canavalia villosa Benth. (EE 19921)	Frijol silvestre	Fodder	Leaves	Fed raw to animals
Dalea bicolor Humb. & Bonpl. ex Willd. var. argyraea (A. Gray) Barneby (EE 16146)	Engorda cabras	Fodder	Leaves	Fed raw to animals

Desmodium grahamii A. Gray (EE 16228)	Pegajosa	Fodder	Whole plant	Fed raw to animals
Eysenhardtia texana Scheele (EE 13325)	Vara dulce	Fodder<br>Treatment of renal diseases<br>Diuretic	Leaves and flowers<br>Leaves<br>Stems and flowers	Fed raw to animals<br>Ingestion of infusion<br>Ingestion of infusion
Indigofera suffruticosa Mill. (EE 13330)	Palo azul	Beverage<br>Remedy for renal problems<br>Dye	Bark<br>Bark<br>Aerial parts	Ingestion of infusion<br>Ingestion of infusion<br>Boiled in water
Lupinus caballoanus B. L. Turner (EE 14682)	Frijolillo	Ornamental	Whole plant	Planted in home gardens
Medicago sativa L. (EE 14872)	Alfalfa	Fodder	Whole plant	Fed raw to animals
Pisum sativum L. (EE 16068)	Chícharo	Food	Seeds	Boiled in soups
Securigera varia (L.) P. Lassen (EE 14666)	Coronilla	Ornamental	Whole plant	Planted in home gardens
Vicia villosa Roth (EE 16087)	Frijol	Fodder	Whole plant	Fed raw to animals
Vigna populnea Piper (EE 16654)	Frijol silvestre	Food<br>Fodder	Seeds<br>Whole plant	Boiled<br>Fed raw to animals
FAGACEAE				
Quercus canbyi Trel. (EE 12729)	Encino	Fodder<br>Construction material<br>Firewood	Acorns<br>Trunk and branches<br>Trunk and branches	Fed raw to animals<br>To build fences and roofs
Quercus coccolobaefolia Trel. (EE 15416)	Encino	Fodder<br>Firewood	Acorns<br>Trunk and branches	Fed raw to animals
Quercus greggii Trel. (EE 16380)	Encino	Fodder	Acorns	Fed raw to animals
Quercus mexicana Humb. & Bonpl. (EE 16303)	Encino	Fodder<br>Firewood	Acorns<br>Trunk and branches	Fed raw to animals
Quercus polymorpha Schltdl. & Cham. (EE 13371)	Encino	Treatment of sensitive gums<br>Remedy for edginess<br>Firewood	Bark<br>Inflorescences<br>Trunk and branches	Mouthwash<br>Ingestion of infusion
Quercus rysophylla Weatherby (EE 13309)	Encino	Fodder<br>Firewood	Acorns	Fed raw to animals
GARRYACEAE				

Garrya ovata Benth. (EE 12724)	Garria	Treatment of chronic diarrhea	Leaves	Ingestion of infusion
GERANIACEAE				
Geranium seemanni Peyr.A.M. (EE 11308)	Geranio, Alfilerillo	Remedy for colic<br>Treatment of throat infections<br>Headaches	Leaves<br>Leaves and stems<br>Leaves (with oil and coffee)	Ingestion of infusion<br>Ingestion of infusion<br>Each side of the head is massaged with a raw leaf
Pelargonium odoratisimum Soland. (EE 16604)	Geranio de olor	Remedy for colic	Leaves	Ingestion of infusion
HYDRANGEACEAE				
Philadelphus madrensis Hemsl. (EE 16112)		Ornamental	Whole plant	Planted in home gardens
JUGLANDACEAE				
Carya myristicaeformis Nutt. (EE 16182)	Nogal cimarrón	Treatment of diabetes<br>Remedy for hair loss<br><br>Colic	Bark<br>Immature fruits<br><br>Leaves	Ingestion of infusion<br>Head is washed with a decoction<br>Ingestion of infusion
Carya ovata K. Koch (EE 16531).	Nogal	Treatment of diabetes<br>Remedy for hair loss<br><br>Colic	Bark<br>Immature fruits<br><br>Leaves	Ingestion of infusion<br>Head is washed with a decoction<br>Ingestion of infusion
Juglans major (Torr.) A. Heller (EE 16471)	Nogal	Treatment of diabetes<br>Remedy for hair loss	Bark<br>Immature fruits	Ingestion of infusion<br>Head is washed with a decoction
LAMIACEAE				
Agastache palmeri (B.L. Rob.) Standl. (EE 16055)	Poleo	Ornamental	Whole plant	Planted in home gardens
Hedeoma costatum A. Gray (JV 7102)	Poleo	Treatment of insomnia; also ingested as a beverage like tea	Aerial parts	Ingestion of infusion
Hedeoma drummondii Benth. (EE 16217)	Poleo	Treatment of insomnia; beverage	Aerial parts	Ingestion of infusion
Hedeoma plicatum Torr. (EE 11322)	Poleo	Treatment of insomnia; beverage	Aerial parts	Ingestion of infusion
Marrubium vulgare Miller. L. (EE 16016)	Marrubio	Treatment of stomach infections<br><br>To stimulate the appetite	Aerial parts, with those of *Artemisia mexicana*<br>Aerial parts	Ingestion of infusion<br><br>Ingestion of infusion

		Remedy for sinus infections<br><br>"Satiated" infant (empachado)<br><br>Treatment of intestinal parasites<br><br>Remedy for colic<br>Treatment of allergies and skin rashes<br><br>"Fright" illness (mal del susto)	Leaves<br><br>Leaves<br><br>Leaves<br><br>Leaves<br>Leaves<br><br>Leaves	Infusion is applied as 2-3 nasal drops three times at day<br>Ground, mixed with maternal milk and fed to infant<br>Toasted and boiled, infusion is ingested<br>Ingestion of infusion<br>Afflicted parts are rubbed with a decoction<br>Ingestion of infusion
Mentha piperita L. (EE 16092)	Menta	Treatment of respiratory problems<br><br>Beverage<br>Remedy for colic	Aerial parts, with *Agastache palmeri*<br>Leaves<br>Leaves	Ingestion of infusion<br><br>Ingestion of infusion<br>Ingestion of infusion
Mentha rotundifolia (L.) Huds. (EE 16073)	Mastranzo	Abortive	Leaves, with *Foeniculum vulgare* and *Quercus polymorpha*	Ingestion of infusion
Mentha spicata L. (EE 16153)	Yerba buena	Childbirth<br><br><br>Treatment of parasites<br>Remedy for stomach problems<br>Flavoring herb<br>Beverage	Aerial parts<br><br><br>Leaves<br>Leaves<br>Aerial parts<br>Aerial parts, with those of *Matricaria chamomilla*	Ingestion of infusion two days after childbirth to avoid hemorrhages<br>Ingestion of infusion<br>Ingestion of infusion<br>Added to broths<br>Ingestion of infusion
Ocimum basilicum L. (EE 18918)	Albácar	Treatment of heart afflictions<br>Remedy for colic<br><br>Flavoring herb	Leaves<br>Aerial parts, with *Agastache mexicana*<br>Aerial parts	Ingestion of infusion<br>Ingestion of infusion<br><br>Added to broths
Rosmarinus officinalis L. (EE 16359)	Romero	Remedy for headaches<br>Treatment of diabetes<br>Rheumatism<br><br>To control blood pressure	Leaves<br>Leaves<br>Leaves<br><br>Leaves	Ingestion of infusion<br>Ingestion of infusion<br>Steeped in alcohol and rubbed on afflicted parts<br>Ingestion of infusion

Salvia ballotaeflora Benth. (EE 16059)	Salvia	Ornamental	Whole plant	Planted in home gardens
Salvia chia Sessé & Moc. (JV 8671)	Chía	Ornamental	Whole plant	Planted in home gardens
Salvia coccinea Juss. ex Murr. (EE 14649)	Betónica	Remedy for headaches<br>To remove debris from the eyes	Stems<br>Seeds	Ingestion of infusion<br>A seed is placed on the cornea: debris sticks to the seed, which is then removed from the eye
Salvia hispanica L. (EE 16742)	Salvia	Remedy for constipation<br>Ornamental	Seeds<br>Whole plant	Ingested like pills<br>Planted in home gardens
Salvia microphylla Sessé & Moc. (EE 14830)	Mirto	Remedy for stomachaches	Leaves	Ingestion of infusion
Salvia regla Cav. (EE 19186)	Salvia	Treatment of anemia (to "swell" the blood)	Leaves	Ingestion of infusion
LAURACEAE				
Litsea pringlei Bartlett (EE 16277)	Laurel	Remedy for heartburn<br><br>Remedy for colic<br>Suffocation<br>Chest pain<br><br>Flavoring herb	Leaves (with *Persea americana* pit)<br>Leaves<br>Leaves<br>Leaves (with *Citrus aurantium* and *Ocimum basilicum*)<br>Aerial parts	Ingestion of infusion<br><br>Ingestion of infusion<br>Ingestion of infusion<br>Ingestion of infusion<br><br>Added to pork
Persea americana Mill. (EE 16788)	Aguacate	Food<br>To "cleanse" the blood<br>Antihelmintic	Fruit<br>Bark<br>Fruit peel	Eaten raw<br>Ingestion of infusion<br>Eaten before breakfast
Persea pachypoda Nees (EE 19707)	Salsafrás	Treatment of anemia (to "swell" the blood)	Leaves and bark (with *Prunus serotina*)	Ingestion of infusion
LILIACEAE				
Allium sativum L. (EE 16370)	Ajo	Control of arterial pressure<br>Earache<br><br><br>Scorpion stings and insects bites	Bulb<br>Bulb<br><br><br>Bulb	A piece of garlic is ingested<br>A piece of garlic is warmed up, wrapped in cotton and inserted into the ear<br>A piece of garlic is rubbed on the sore skin

		Food<br><br>Condiment	Bulb<br><br>Bulb	Eaten raw and cooked in broths and with beans<br>Added to foods and dressings
Aloe vera L. (EE 14908)	Sábila	Remedy for toothache<br><br>To heal wounds<br>Inflammation<br>Treatment of acne<br><br><br><br>Shampoo<br><br>Treatment of diabetes<br><br>Remedy for gastritis<br><br>Treatment of circulatory ailments<br><br>Treatment of cancer<br>Anemia<br>Arthritis<br>To remove skin blots	Leaves<br><br>Pulp of the leaves<br>Pulp of the leaves<br>Pulp of the leaves<br><br><br><br>Leaves<br><br>Leaves<br><br>Leaves<br><br>Leaves<br><br>Raw pulp<br>Raw pulp<br>Raw pulp<br>Leaves	Wrapped in cloth, applied on afflicted area<br>Applied as a poultice<br>Applied as a poultice<br>Blended and smeared on the face 2-3 times a week; treatment strengthens and softens the skin<br>Blended in water, used as a hair wash<br>Blended in water, ingested before breakfast<br>Blended in water with orange juice and ingested<br>Blended in water, ingested before breakfast<br>Eaten before breakfast<br>Eaten before breakfast<br>Eaten before breakfast<br>The juice of the pulp is applied to the stained skin
Schoenocaulon texanum Scheele (EE 16339)	Cola de zorra	Ornamental	Whole plant	Planted in home gardens
MALPIGHIACEAE				
Mascagnia macroptera Nied. (EE 16201)	Piñata	Ornamental	Whole plant	Planted in home gardens
MALVACEAE				
Hibiscus cardiophyllus A. Gray (EE 19704)	Tulipán	Ornamental	Whole plant	Planted in home gardens
Hibiscus coulteri Harv. ex A. Gray (EE 16172)	Tulipán amarillo	Ornamental	Whole plant	Planted in home gardens
MIMOSACEAE				

Acacia farnesiana (L.) Willd. (EE 19370)	Huizache	Fodder<br>Firewood<br>Remedy for dyspepsia<br>Treatment of dysentery<br>Vaginal hemorrhages	Leaves, fruits<br>Trunk and branches<br>Flowers<br>Fruits<br>Roots	Fed raw to animals<br><br>Ingestion of infusion<br>Boiled and ingested as infusion<br>Boiled
Acacia greggii A. Gray. (EE 13387)	Uña de gato	Treatment of cancer	Aerial parts	Ingestion of infusion
Acacia rigidula Benth. (EE 13333)	Chaparro prieto	Fodder<br>Firewood	Leaves<br>Trunk and branches	Fed raw to animals
Ebenopsis ebano (Berland.) Barneby & J.W. Grimes (EE 16231)	Ébano	Handicrafts<br>Food<br>Beverage<br><br>Carpentry	Wood<br>Seeds<br>Seeds<br><br>Wood	Carved<br>Boiled<br>Toasted, ground and boiled as a substitute for coffee<br>Made into furniture
Havardia pallens Britt & Rose (EE 14645)	Tenaza	Fodder<br>Construction material	Leaves<br>Trunk	Fed raw to animals<br>To build fences, roofs, walls
Leucaena greggii S. Watson (EE 16104)	Tepehuaje	Fodder	Leaves	Fed raw to animals
Leucaena leucocephala (Lam.) DeWit ssp. *glabrata* (Rose) S. Zárate (EE 16049)	Dormilón	Fodder	Leaves	Fed raw to animals
Prosopis glandulosa Torr. var. *glandulosa* (EE 13337)	Mezquite	Fodder<br>Construction material<br>Food<br>Firewood	Leaves and fruit pods<br>Trunk<br>Fruit pods<br>Trunk and branches	Fed raw to animals<br>To build fences, roofs, walls<br>Eaten raw
MORACEAE				
Ficus carica L. (EE 16164)	Higo	Food	Infrutescence	Eaten raw or cooked and canned
Morus celtidifolia Kunth (EE 13381)	Mora	Treatment of diabetes<br>Food	Fruit<br>Fruit	Ingestion of decoction<br>Eaten raw
NOLINACEAE				
Dasylirion texanum Scheele (EE 16180)	Sotol	Thatching material for roofs<br>Alcoholic beverage	Leaves<br>Sap	Tied into bundles<br>Fermented and distilled
Hesperaloe funifera Trel.	Sotol	Fiber	Leaves	Scraped and twisted into

(EE 19475)				rope
NYCTAGINACEAE				
Bougainvillea glabra Choisy (EE 18934)	Bugambilia	Cough remedy<br>Ornamental	Bracts and flowers<br>Whole plant	Ingestion of infusion<br>Planted in home gardens
Mirabilis jalapa L. (EE 16199)	Maravilla	Treatment of epilepsy<br>Purgative; anticrotalic; renal and hepatic ailments	Flowers<br>Leaves	Ingestion of infusion<br>Ingestion of infusion
ORCHIDACEAE				
Corallorhiza maculata Greene (EE 16262)	Orquídea	Ornamental	Whole plant	Planted in home gardens
Dichromanthus cinnabarinus (La Llave & Lex.) Garay (EE 16488)	Orquídea	Ornamental	Whole plant	Planted in home gardens
Hexalectris revoluta Correll	Orquídea	Ornamental	Whole plant	Planted in home gardens
Malaxis macrostachya Kuntze (EE 16698)	Orquídea	Ornamental	Whole plant	Planted in home gardens
Malaxis corymbosa Kuntze (16640)	Orquídea	Ornamental	Whole plant	Planted in home gardens
Platanthera sparsiflora Schltr. (EE 16302)	Orquídea	Ornamental	Whole plant	Planted in home gardens
Spiranthes vernalis Engelm. & A. Gray (EE 16137)	Orquídea	Ornamental	Whole plant	Planted in home gardens
PHYTOLACCACEAE				
Phytolacca icosandra L. (EE 16268)	Fitolaca	Remedy for dandruff	Leaves	The hair is washed with a solution of the boiled leaves
PINACEAE				
Pinus cembroides Gordon (EE 19558)	Pino	Firewood<br>Construction material	Trunk and branches<br>Trunk	Burnt in ovens<br>To build houses, fences, roofs
Pinus greggii Engelm. ex Parl. (EE 12731)	Pino	Firewood<br>Construction material	Trunk and branches<br>Trunk	Burnt in ovens<br>To build houses, roofs, fences
Pinus pseudostrobus Lindl. (EE 11281)	Pino	Treatment of bronchitis<br>Firewood<br>Construction material	Leaves<br>Trunk and branches<br>Trunk	Ingestion of infusion<br>Burnt in ovens<br>Houses, roofs, fences, etc.
Pinus teocote Cham. & Schltdl. (EE 11803)	Ocote	Firewood<br>Construction material	Trunk and branches<br>Trunk	Burnt in ovens<br>Houses, roofs, fences, etc.

PLANTAGINACEAE				
Plantago major L. (EE 16190)	Lengua de vaca	Antibiotic, antiinflammatory	Leaves	Boiled, ingestion of infusion
POACEAE				
Avena sativa L. (EE 16096)	Avena	Treatment of gastritis<br><br>Food	Raw seeds<br><br>Seeds	Soaked in water for one day, blended and ingested as a drink during 9 days<br>Made into a beverage, raw or cooked
Cymbopogon citratus Stapf. (EE 18968)	Zacate limón	Beverage	Leaves	Ingestion of infusion
Zea mays L. (EE 16288)	Maíz, elote	Food<br><br>Animal fodder<br>Treatment of cough, dysentery; bladder inflammations, diabetes and menstrual colic	Infrutescence (mazorca)<br><br>Aerial parts<br>Corn "hairs" (styles)	Made into tortillas; tender ears are boiled or roasted, tender seeds are fried (esquite), also added to soups and broths<br>Fed raw to animals<br>Ingestion of infusion, at times with honey
POLYGONACEAE				
Rumex mexicanus Meisn. (EE 16097)	Acelga	Food	Tender leaves	Eaten raw in salads, boiled in soups or mixed with eggs, tomatoes and onions
Rumex pulcher L. (EE 14661)	Lengua de vaca	Food<br><br>Treatment of vaginal infections<br><br>Fodder	Tender leaves<br><br>Leaves<br><br>Aerial parts	Eaten raw in salads, boiled in soups or mixed with eggs, tomatoes and onions<br>Infusion ingested and applied as a vaginal wash<br>Fed raw to animals
PORTULACACEAE				
Portulaca oleracea L. (EE 16699)	Verdolaga	Food	Aerial parts	Eaten raw in salads and cooked
PTERIDACEAE				
Pteridium aquilinum (L.) Kuhn (EE 16181)	Zarzaparrilla	Remedy for colic	Roots	Ingestion of infusion
PUNICACEAE				

Punica granatum L. (EE 11211)	Granada	Food<br>Remedy for diarrhea	Fruits<br>Flowers (with *Psidium guajava* flowers)	Eaten raw<br>Ingestion of infusion
RANUNCULACEAE				
Clematis dioica L. (EE 16441)	Barba de chivo	Pain relief for toothache	Stem	A piece of raw stem is applied to the afflicted area
Clematis drummondii Torr. & A. Gray (EE 14420)	Barba de chivo	Toothache	Stem	A piece of raw stem is applied locally
ROSACEAE				
Cowania plicata D. Don (EE 12733)	Alejandría	Remedy for colic and stomachaches	Leaves	Ingestion of infusion
Crataegus tracyi Ashe (EE 16054)	Tejocote	Food<br><br>Treatment of diabetes	Fruit<br><br>Fruit peel	Eaten raw or cooked and canned<br>Ingestion of infusion
Eriobotrya japonica (Thunb.) Lindl. (EE 10911)	Níspero	Remedy for colic	Leaves and fruit peel	Ingestion of infusion
Fragaria mexicana Schltdl. (EE 14671)	Fresa silvestre	Food	Fruit	Eaten raw
Malus domestica Borkh. (EE 11466)	Manzana	Food	Fruit	Eaten raw or cooked and canned; also made into cider
Prunus domestica L. (EE 16165)	Ciruelo	Food	Fruit	Eaten raw or cooked and canned
Prunus persica (L.) Batsch (EE 19249)	Durazno	To remove intestinal parasites<br>Food	Leaves<br>Fruit	Ingestion of infusion<br>Eaten raw or cooked and canned
Prunus serotina Poir. (EE 16515)	Capulín, quina	Treatment of anemia<br>Abortive<br>Liqueur	Bark<br>Leaves and bark<br>Fruit	Ingestion of infusion<br>Ingestion of infusion<br>Fermented
Rosa serrulata Raf. (EE 16133)	Rosa	Remedy for constipation<br>Colic<br>Diarrhea<br>To stop vomiting	Flowers<br>Flowers<br>Flowers<br>Flowers	Ingestion of infusion<br>Ingestion of infusion<br>Ingestion of infusion<br>Ingestion of infusion
Vauquelinia corymbosa Correa ex Humbl. & Bonpl. (EE 12739)	Saucillo	Ornamental	Whole plant	Planted in home gardens
RUBIACEAE				

Cephalanthus salicifolius Humbl. & Bonpl. (EE 16572)	Sauz	Laxative<br>Treatment of venereal diseases	Leaves<br>Bark	Boiled, ingestion of infusion<br>Ingestion of infusion
Chiococca alba Rusby (JV 8674)	Perlas de la Virgen	Remedy for stomachaches, renal pains, diabetes	Leaves	Ingestion of infusion
RUTACEAE				
Casimiroa greggii (S. Watson) F. Chiang (EE 16211)	Manguito	To control arterial pressure	Leaves	Igestion of infusion
Esenbeckia berlandieri Baill. (EE 16259)	Manguito	Ornamental	Whole plant	Planted in home gardens
Helietta parvifolia Benth. (EE 16253)	Barreta	Firewood<br>Construction material<br>Treatment of rheumatism	Trunk and branches<br>Trunk<br>Leaves	Burnt in ovens<br>To build houses, roofs, fences<br>Ingestion of infusion
Ruta graveolens L. (EE 16015)	Ruda	Remedy for chest pains<br>Earaches<br><br>Headaches<br><br>Abortive	Leaves<br>Leaves<br><br>Leaves (with oil)<br><br>Leaves (with 9 seeds of *Phaseolus vulgaris* and *Bidens odorata*)	Ingestion of infusion<br>Raw leaves are crushed and inserted into the ear<br>Leaves are adhered to the sides of the head<br>Ingestion of infusion
SAPINDACEAE				
Dodonaea viscosa Mart. (EE 13304)	jara	Remedy for colic; treatment for sterility	All parts	Boiled and ingested
SAXIFRAGACEAE				
Heuchera mexicana W. Schaffn. (EE 16108)	Saxifraga	Ornamental	Whole plant	Planted in home gardens
SELAGINELLACEAE				
Selaginella lepidophylla (Hook. et A. Gray) Spring (EE 16688)	Siempreviva	Treatment of renal ailments, diarrhea, bronchitis	Leaves	Ingestion of infusion
Selaginella pilifera A. Br. (EE 16273)	Siempreviva	Treatment of renal ailments	Leaves	Ingestion of infusion
SMILACACEAE				

Smilax bona-nox L. (EE 16233)	Zarzaparrilla	To lose weight<br>Treatment of circulatory problems, anemia, renal ailments<br>Dysentery	Roots<br>Leaves<br><br>Roots	Ingestion of infusion<br>Ingestion of infusion<br><br>Ingestion of infusion
Smilax moranensis M. Martens & Galeotti. (EE 19341)	Cocolmeca	Remedy for renal pain	Roots	Ingestion of infusion
SOLANACEAE				
Datura stramonium Wall. (EE 16776)	Toloache	Treatment of wounds	Leaves	Applied with oil as a poultice
Lycopersicon esculentum Mill. (EE 12981).	Tomate	Food<br>Remedy for cramps	Fruit<br>Fruit peel	Raw edible<br>Boiled, ingestion of infusion
Nicandra physaloides Gaertn. (EE 19429)	Belladona	Treatment of urinary problems of livestock	Leaves	Boiled in 2-3 liters of water and given to animals to drink
Physalis pringlei Greenm. (EE 19225)	Tomatillo	Remedy for cramps<br>Food	Fruit husk<br>Fruit	Ingestion of infusion<br>Eaten raw or cooked in sauces
Physalis stapelioides (Regel) Bitter. (EE 16351)	Tomatillo	Food	Fruit	Eaten raw or cooked in sauces
Solanum erianthum D. Don (EE 16234)	Malabar	Treatment of diabetes	Leaves	Ingestion of infusion
TAXACEAE				
Taxus globosa Schltdl., (EE 19436)	Chiper	Firewood<br>Construction material<br>Remedy for stomachaches<br>Headaches	Trunk and branches<br>Trunk<br>Leaves<br>Leaves (with *Taraxacum officinale* and *Ruta graveolens* leaves)	Burnt in ovens<br>To build houses, roofs, fences<br>Ingestion of infusion<br>Ingestion of infusion
TAXODIACEAE				
Taxodium mucronatum Ten. (EE 16299)	Sabino	Remedy for diarrhea<br>Construction material	Leaves<br>Trunk and branches	Ingestion of infusion<br>To build houses, fences
UMBELLIFERAE				
Foeniculum vulgare Mill. (EE 13093)	Hinojo	Remedy for colic	Leaves	Ingestion of infusion

VERBENACEAE				
Glandularia bipinnatifida Nutt. (EE 16011)	Verbena	Treatment of boils	Aerial parts, applied to the afflicted area	Infusion
Lantana camara L. (EE 14885)	Mejorana	Ornamental	Whole plant	Planted in home gardens
Lantana macropoda Torr. (EE 16218)	Mejorana	Remedy for stomachaches<br>Back pain<br>Treatment of amoebic dysentery<br>Remedy for toothache<br>Diuretic	Leaves<br>Leaves (with *Scutellaria microphylla* and *Bidens odorata*)<br>Roots and leaves<br>Roots and leaves<br>Leaves	Ingestion of infusion<br>Ingestion of infusion<br>Infusion ingested as a tea<br>Ingestion of infusion<br>Ingestion of infusion
VITACEAE				
Vitis berlandieri Planch. (EE 16364)	Parra	Remedy for colic<br>Treatment of eye infections	Stems and leaves<br>Leaves	Ingestion of infusion<br>Infusiona pplied as eye drops
ZYGOPHYLLACEAE				
Larrea tridentata Coult. (EE 18456)	Gobernadora	Treatment of renal ailments<br>Digestive disorders	Leaves<br>Leaves	Ingestion of infusion<br>Ingestion of infusion
